# Terahertz magnetic response of plasmonic metasurface resonators: origin and orientation dependence

**DOI:** 10.1038/s41598-024-65804-9

**Published:** 2024-07-03

**Authors:** Lorenzo Tesi, Martin Hrtoň, Dominik Bloos, Mario Hentschel, Tomáš Šikola, Joris van Slageren

**Affiliations:** 1 Institute of Physical Chemistry, University of Stuttgart, Pfaffenwaldring 55, 70569 Stuttgart, Germany; 2 Institute of Physical Engineering and Central European Institute of Technology, Brno University of Technology, Technická 2, 61669 Brno, Czech Republic; 3 4th Physics Institute and Research Center SCoPE, University of Stuttgart, Pfaffenwaldring 57, 70569 Stuttgart, Germany; 4 Center for Integrated Quantum Science and Technology, University of Stuttgart, Stuttgart, Germany

**Keywords:** Magnetic metasurface, Terahertz, Cavity-enhanced, Fabry-Pérot, Electron paramagnetic resonance, Metamaterials, Photonic devices

## Abstract

The increasing miniaturization of everyday devices necessitates advancements in surface-sensitive techniques to access phenomena more effectively. Magnetic resonance methods, such as nuclear or electron paramagnetic resonance, play a crucial role due to their unique analytical capabilities. Recently, the development of a novel plasmonic metasurface resonator aimed at boosting the THz electron magnetic response in 2D materials resulted in a significant magnetic field enhancement, confirmed by both numerical simulations and experimental data. Yet, the mechanisms driving this resonance were not explored in detail. In this study, we elucidate these mechanisms using two semi-analytical models: one addressing the resonant behaviour and the other examining the orientation-dependent response, considering the anisotropy of the antennas and experimental framework. Our findings contribute to advancing magnetic spectroscopic techniques, broadening their applicability to 2D systems.

## Introduction

**Figure 1:**
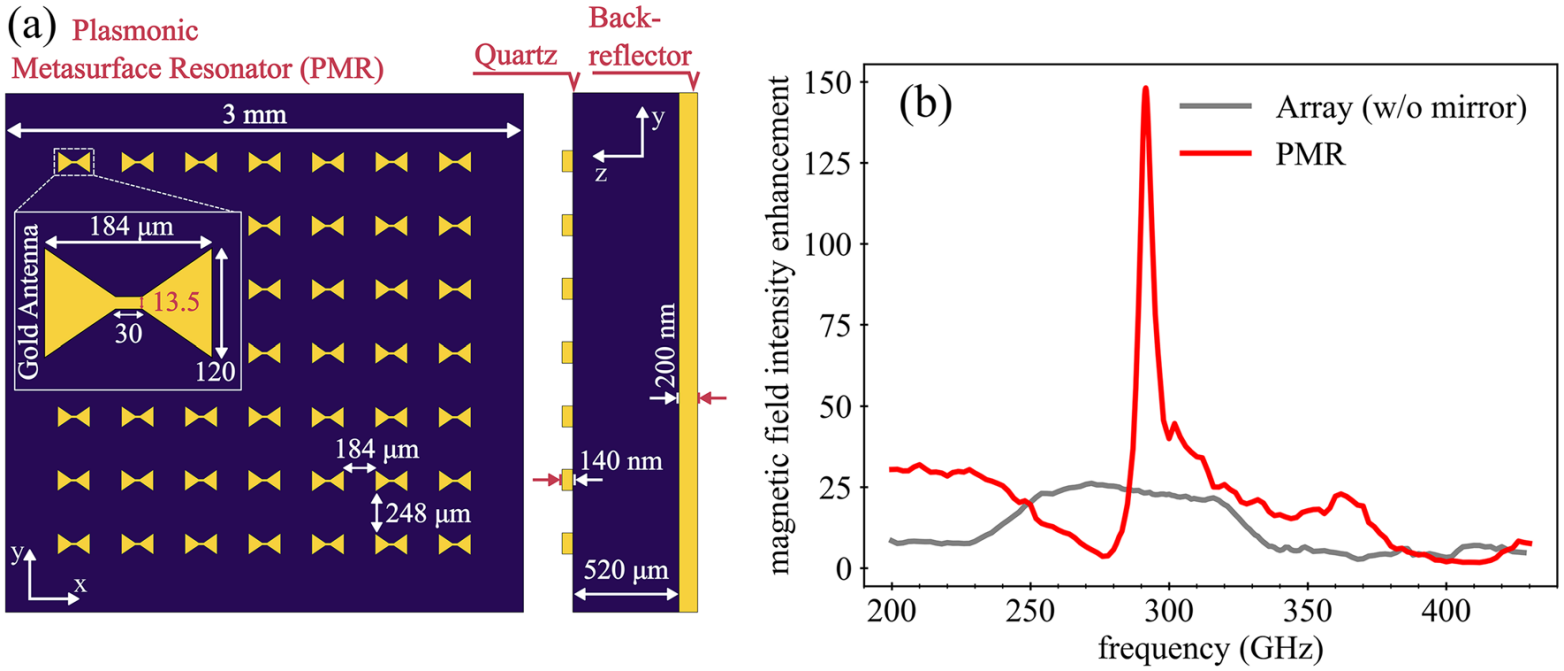
(**a**) Design parameters of the PMR. (**b**) Resonance intensity of the in-plane magnetic field intensity enhancement of the PMR (array and back-reflector) and array without back-reflector obtained by numerical simulations. The near-field intensities were determined 10 nm above the antennas top plane and rescaled by the source intensity to obtain the field enhancement. More details in reference^23^.

The study of thin layers, surface-immobilized species, and volume-limited samples is pivotal across multiple disciplines in physics, chemistry, biology and material sciences. These investigations include the challenge of analysing biomolecules that are available only in limited quantities^1^, magnetic materials with properties influenced by their dimensionality^2–4^, inorganic and organic semiconductors of interest for spintronics^5–8^, and thin layers of magnetic materials appealing for quantum technologies^9–14^. Each of these topics requires highly sensitive and surface-specific techniques. Electron paramagnetic resonance (EPR) spectroscopy has emerged as a key technique with the potential to advance research across these scientific areas. This is a powerful analytical tool that explores the magnetic properties of electron spins in materials under the influence of an external magnetic field. Widely applicable to any system with unpaired electrons, this technique provides crucial insights into the electronic structures and dynamic behaviours at the microscopic level. While recent advances have significantly improved the sensitivity of EPR at frequencies below 9 GHz^15–18^, scaling up these successes to the THz range (> 100 GHz) remains a significant challenge. The use of THz frequencies offers unprecedented advantages over conventional EPR techniques, which typically operate at either 9 or 35 GHz, in terms of resolution and access to larger energy splittings, allowing more comprehensive analysis of a wide range of phenomena^19–22^. Recent advances in THz EPR resonators have opened up new possibilities in overcoming these inherent challenges associated with the need for excitation of magnetic transitions, which are weaker compared to electric ones. In particular, we have recently realized novel resonators based on plasmonic metasurfaces^23^. Generally, plasmonic metasurfaces, comprised of artificially structured nanoantennas, enable unprecedented control over electromagnetic waves, offering new avenues in manipulating light at the subwavelength scale^24^. Similar control of surface plasmon waves can also be achieved with plasmonic grooves, which consist of periodic nanoscale indentations, and waveguide structures^25,26^. These innovative plasmonic structures have paved the way for a myriad of applications, including enhanced biosensing^27^, improved optical devices^28^, and the development of highly efficient energy harvesting systems^29^. However, most of the plasmonic metasurface structures designed so far are based on the manipulation of the electric field component of the radiation, whereas to enhance magnetic transitions in EPR spectroscopy, only the magnetic component is important. With the aim of confining the magnetic field to the surface to enhance the magnetic response of 2D materials, in a previous work we have realized a plasmonic metasurface resonator (PMR) consisting of an array of gold diabolo-like antennas fabricated on a quartz substrate with a metallic back-reflector (Fig. 1a)^23^. A strong magnetic field intensity enhancement at around 290 GHz with quality (Q-) factor of 40 was demonstrated both by simulations and experiments (Figs. 1b and S1). Any deviation from the geometric parameters used for the resonator design results in a slight decrease in the resonance amplitude. However, a dramatic decrease was observed when the metal back-reflector is removed (Fig. 1b). The precise origin of such a strong resonance was thus far not really understood, although this is pivotal to progress in the realization of more efficient resonators. In addition, the orientation dependence of the response of the PMR was previously not investigated, despite the fact that the anisotropic nature of the antennas can lead to different responses depending on the polarization of the incident radiation. These two issues, namely (i) understanding the origin of the PMR resonance and (ii) its orientation-dependent response, were a critical knowledge gap in exploiting the full potential of PMRs for THz applications. Therefore, they are the subject of the first and second parts of this article, respectively. To address them, we have developed two semi-analytical models that explain the experimental observations. The results indicate that the strong resonance of the PMR is attributed to the interplay between the plasmonic excitation of the antenna and the standing waves formed in the substrate, which is driven by the back-reflector. The model effectively elucidates the dynamic interaction at play, revealing how the antenna array, paired with the back-reflector, creates a high-finesse cavity through a feedback loop mechanism. Furthermore, our analysis unveils an optimal THz EPR signal enhancement at a specific polarization angle, challenging previous assumptions about resonator activity and orientation. Therefore, this study represents a step forward in both theoretical understanding and practical application of cavity-enhanced plasmonic structures as well as THz magnetic metasurfaces.

## Results and discussion

### Origin of the resonance

**Figure 2:**
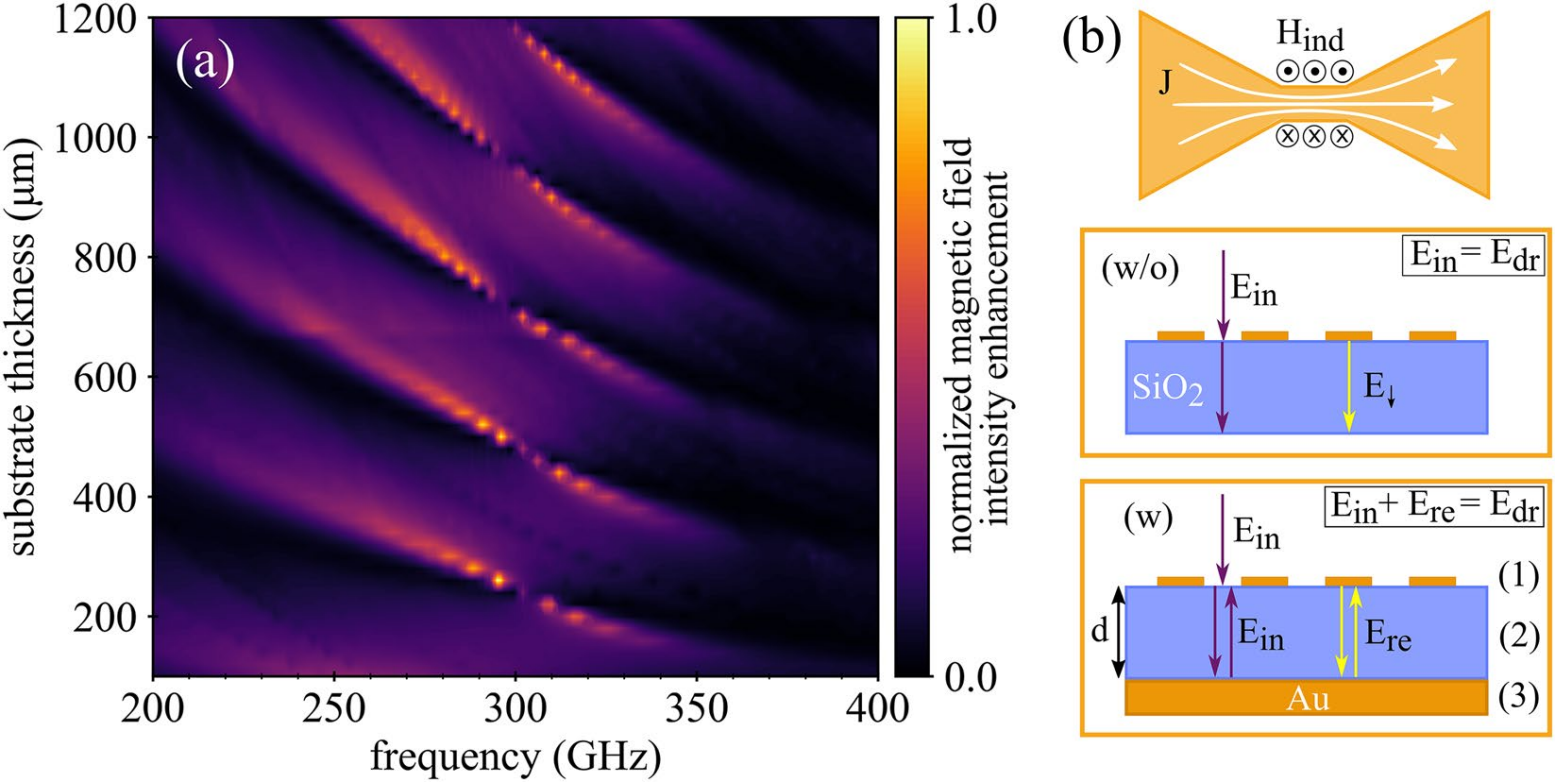
(**a**) Map of normalized in-plane magnetic field intensity enhancement of the PMR simulated as a function of frequency and substrate thickness. The simulation was performed with CST Microwave Studio using the parameters in Fig. 1a. (**b**) Scheme and parameters used in the semi-analytical model (see text).

The starting point for our investigation into the mechanism responsible for the sharp spectral response observed in the PMR was numerical simulations performed using CST Microwave Studio: The PMR response was analysed by sweeping over the various geometrical parameters of antennas and substrate. Sweeping over the substrate thickness revealed a regular pattern of high intensity fringes in the 200–400 GHz frequency range (Fig. 2a). Interestingly, all the fringes are interrupted by a sudden gap at ca. 300 GHz, with the highest magnetic field intensity enhancement occurring at a slightly lower frequency. The existence of regularly spaced branches is indicative of Fabry-Pérot oscillations arising between the top and bottom faces of the quartz substrate. However, this effect still does not explain the origin of the gap at 300 GHz. The fact that its spectral position can be tuned by changing the antenna geometrical parameters (Fig. S2) indicates that the optical response of the antenna array plays a role and that Fabry-Pérot oscillations alone are not enough to satisfactorily explain all the features present in Fig. 2a.

To clarify the origin of the fringe splitting at 300 GHz, we developed a semi-analytical model that treats the antenna array as a single collective unit that is driven by (i) an external electric field comprising both the incident wave and (ii) the wave reflected from the mirror covering the bottom side of the substrate, i.e. the back-reflector. The only external input (iii) to our model is the optical response of the antenna array, which was calculated numerically using Ansys/Lumerical FDTD. Importantly, the quartz/gold interface introduced by the back-reflector was not included explicitly in these simulations. Instead, it enters the model analytically through Fresnel coefficients describing all the various reflections occuring in our multilayer system. Using this approach, one can identify the resonance condition for the Fabry-Pérot mode inside the quartz substrate, including the role played by the antenna array. Clearly, our model is based on the assumption that by adding the quartz/gold interface, one does not appreciably change the optical response of the antenna array, i.e. the distribution of currents $\vec{j}\left(\vec{r},\omega \right)$ induced within the antennas remains—apart from a scaling factor—the same. Mathematically, this translates to

1 $$\vec{j}\left( {\vec{r},\omega } \right) = j\left( \omega \right) \vec{j}_{0} \left( {\vec{r},\omega } \right),$$

where ${\vec{j}}_{0}\left(\vec{r},\omega \right)$ represents the fixed spatial mode distribution and $j\left(\omega \right)$ its excitation amplitude. The latter captures all the effects associated with the introduction of the quartz/gold interface, including Fabry-Pérot oscillations and their supression for a certain frequency leading to the splitting of the fringes. A thorough analysis (see supplementary information) confirmed the validity of this approximation within the range of paramaters considered in this study (Fig. S3). Finding a concise and insightful expression for the excitation amplitude $j\left(\omega \right)$ is therefore the main goal of the following derivation.

Let us start by considering the relation between the magnetic field intensity enhancement $\eta \left(\omega \right)$ and the excitation amplitude $j\left(\omega \right)$. Close to the antenna array, the induced magnetic field ${\vec{H}}_{\text{ind}}$ generated by $\vec{j}\left(\vec{r},\omega \right)$ represents the dominant contribution to the magnetic field intensity enhancement and scaling of the excitation amplitude $j\left(\omega \right)$, therefore, directly affects it:

2 $$\eta \left( \omega \right) \sim \left| {\vec{H}_{{{\text{ind}}}} \left( {\vec{r},\omega } \right)} \right|^{2} \sim \left| {j\left( \omega \right)} \right|^{2} .$$

Similarly, the excitation amplitude $j\left(\omega \right)$ is directly proportional to the electric field driving the antenna array, ${E}_{\text{dr}}\left(\omega \right)$, via the polarizability factor $\alpha \left(\omega \right)$.

3 $$j\left( \omega \right) = \alpha \left( \omega \right) E_{{{\text{dr}}}} \left( \omega \right).$$

Since we assume that the optical response of the antenna array—here captured by $\alpha \left(\omega \right)$—remains the same even after the introduction of the quartz/gold interface, any change in the excitation amplitude must come from the driving field ${E}_{\text{dr}}\left(\omega \right)$. Note that the driving field includes not only the illuminating wave, but also all the waves previously emitted by the antenna array and subsequently scattered by its surroundings. Assigning the superscripts (w) and (w/o) to the two situations with and without the quartz/gold interface, respectively, one obtains

4 $$\frac{{j^{{\left( {\text{w}} \right)}} \left( \omega \right)}}{{j^{{\left( {{\text{w}}/{\text{o}}} \right)}} \left( \omega \right)}} = \frac{{E_{{{\text{dr}}}}^{{\left( {\text{w}} \right)}} \left( \omega \right)}}{{E_{{{\text{dr}}}}^{{\left( {{\text{w}}/{\text{o}}} \right)}} \left( \omega \right)}}.$$

While ${E}_{\text{dr}}^{(\text{w}/\text{o})}\left(\omega \right)$ simply corresponds to the amplitude of the incident field, ${E}_{\text{dr}}^{(\text{w})}\left(\omega \right)$ incorporates also the wave that is reflected back from the quartz/gold interface after its emission from the antenna array. Designating the complex amplitude of this back-reflected wave as ${E}_{\text{re}}^{(\text{w})}\left(\omega \right)$, which can be seen as the array feedback factor, the above equation can be recast as

5 $$\frac{{j^{{\left( {\text{w}} \right)}} \left( \omega \right)}}{{j^{{\left( {{\text{w}}/{\text{o}}} \right)}} \left( \omega \right)}} = \frac{{E_{{{\text{in}}}}^{{\left( {\text{w}} \right)}} \left( \omega \right) + E_{{{\text{re}}}}^{{\left( {\text{w}} \right)}} \left( \omega \right)}}{{E_{{{\text{in}}}}^{{\left( {{\text{w}}/{\text{o}}} \right)}} \left( \omega \right)}},$$

where ${E}_{\text{in}}^{(\text{w}/\text{o})}\left(\omega \right)$ and ${E}_{\text{in}}^{(\text{w})}\left(\omega \right)$ stand for the amplitudes of the incident field at the position of the antenna array. Note that those two quantities generally differ as the quartz/gold interface affects also the illuminating wave by reflecting it. Assuming topside illumination under normal incidence and marking the spaces above, inside, and below the substrate with indices 1, 2, and 3 (cf. Figure 2b), the complex amplitudes ${E}_{\text{in}}^{(\text{w}/\text{o})}\left(\omega \right)$ and ${E}_{\text{in}}^{(\text{w})}\left(\omega \right)$ can be expressed as

6 $$E_{{{\text{in}}}}^{{\left( {{\text{w}}/{\text{o}}} \right)}} \left( \omega \right) = 1 - r_{12} ,$$

7 $$E_{{{\text{in}}}}^{{\left( {\text{w}} \right)}} \left( \omega \right) = 1 - \frac{{r_{12} + r_{23} e^{{2ik_{2} d}} }}{{1 - r_{12} r_{23} e^{{2ik_{2} d}} }},$$

with ${r}_{12}$ and ${r}_{23}$ representing Fresnel reflection coefficients at the interfaces between the respective media, ${k}_{2}$ standing for the wavenumber inside the substrate, and $d$ denoting the substrate thickness. The last piece of the puzzle that remains to be specified is the reflected electric field amplitude ${E}_{\text{re}}^{(\text{w})}\left(\omega \right)$. In our particular case, it depends on the amplitude ${E}_{\downarrow }^{(\text{w}/\text{o})}\left(\omega \right)$ of the wave that the antenna array emits into the substrate in the absence of the quartz/gold interface (and which is supplied by FDTD simulations) according to:

8 $$E_{{{\text{re}}}}^{{\left( {\text{w}} \right)}} \left( \omega \right) = \frac{{j^{{\left( {\text{w}} \right)}} \left( \omega \right)}}{{j^{{\left( {{\text{w}}/{\text{o}}} \right)}} \left( \omega \right)}} E_{ \downarrow }^{{\left( {{\text{w}}/{\text{o}}} \right)}} \left( \omega \right) \frac{{t_{21} r_{23} e^{{2ik_{2} d}} }}{{1 - r_{12} r_{23} e^{{2ik_{2} d}} }}.$$

**Figure 3:**
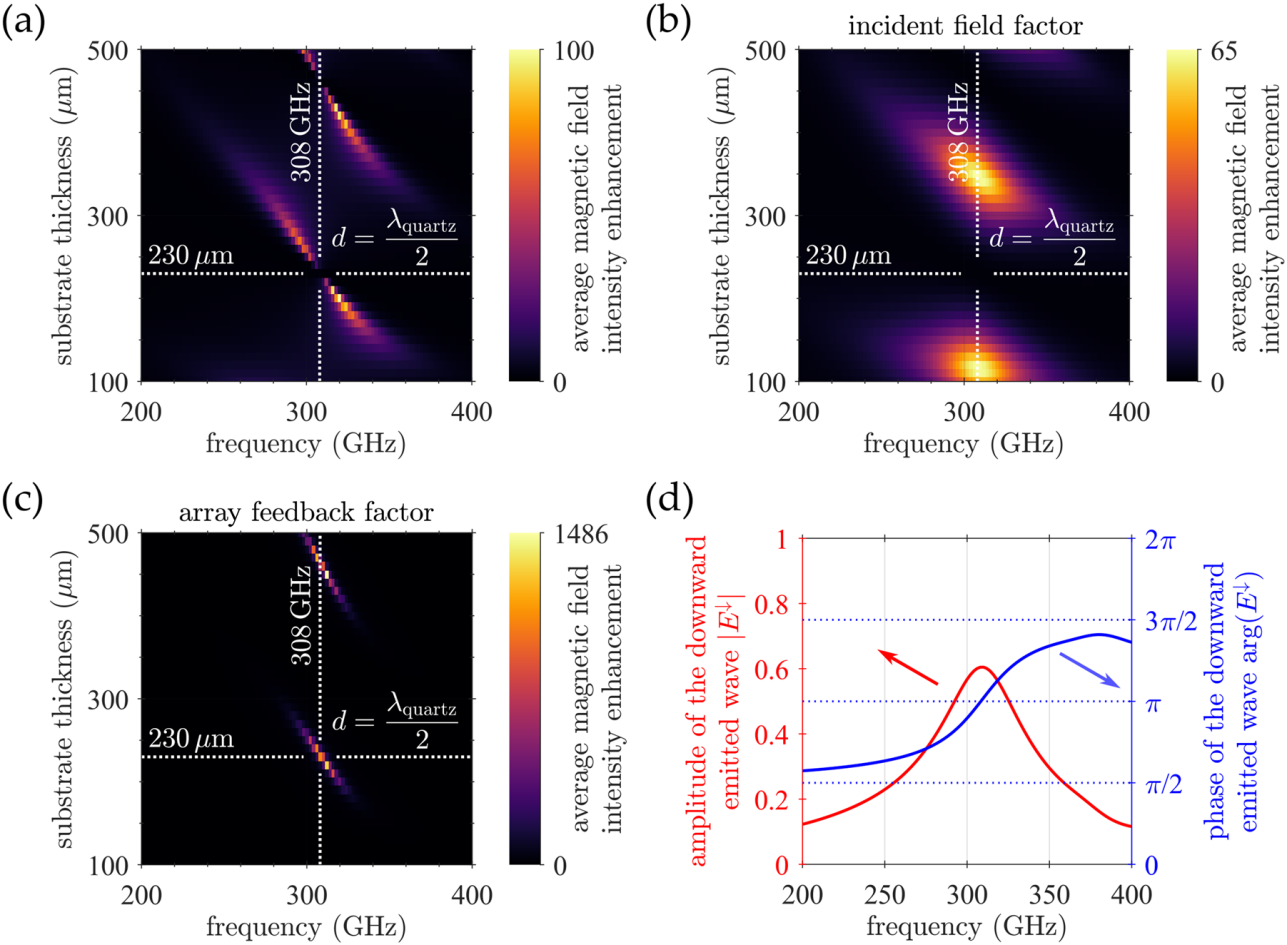
(**a**) Map of the average magnetic field intensity enhancement as a function of frequency and substrate thickness calculated by our semi-analytical model, which reproduces the dispersion branches observed in Fig. 2. (**b**) Map of the average magnetic field intensity enhancement based solely on the incident field factor incorporating only the effect of the quartz/gold interface on the illuminating wave. (**c**) Map of the average magnetic field intensity enhancement utilizing only the array feedback factor (i.e., ${E}_{\text{re}}^{(w)}\left(\omega \right)$), which captures the interaction of the array with itself. The intersection of the white dotted lines in all the maps marks the gap in the dispersion branch due to the complete destructive interference between the forward propagating incident wave and its back-reflected counterpart. (**d**) Amplitude and phase of the wave emitted into the quartz substrate by the antenna array as a function of frequency obtained by FDTD simulations.

Here the Fresnel coefficient ${t}_{21}$ describes transmission through the quartz/air interface, while the factor ${j}^{(\text{w})}\left(\omega \right)/{j}^{(\text{w}/\text{o})}\left(\omega \right)$ needs to be included in order to ensure proper scaling of the radiation emission from the antenna array with respect to the situation without the quartz/gold interface. Finally, by combining Eqs. (5–8), one obtains the sought closed form expression for the ratio of the excitation amplitudes

9 $$\frac{{j^{{\left( {\text{w}} \right)}} \left( \omega \right)}}{{j^{{\left( {{\text{w}}/{\text{o}}} \right)}} \left( \omega \right)}} = \frac{{\left( {1 - \frac{{r_{12} + r_{23} e^{{2ik_{2} d}} }}{{1 - r_{21} r_{23} e^{{2ik_{2} d}} }}} \right)/\left( {1 - r_{12} } \right)}}{{1 + \frac{{E_{ \downarrow }^{{\left( {{\text{w}}/{\text{o}}} \right)}} \left( \omega \right)}}{{1 - r_{12} }}\frac{{t_{21} r_{23} e^{{2ik_{2} d}} }}{{1 - r_{21} r_{23} e^{{2ik_{2} d}} }}}},$$

which fully captures—as we shall demonstrate in the following analysis—the peculiar branch splitting encountered in Fig. 2. To recreate that intensity map with our semi-analytical model, we should deal with the magnetic field intensity enhancement instead of the excitation amplitude. Recalling the relation between $\eta \left(\omega \right)$ and $j\left(\omega \right)$ given by Eq. (2), the magnetic field intensity enhancement ${\eta }^{(\text{w})}\left(\omega \right)$ in the presence of the quartz/gold interface reads

10 $$\eta^{{\left( {\text{w}} \right)}} \left( \omega \right) = \left| {\frac{{j^{{\left( {\text{w}} \right)}} \left( \omega \right)}}{{j^{{\left( {{\text{w}}/{\text{o}}} \right)}} \left( \omega \right)}}} \right|^{2} \eta^{{\left( {{\text{w}}/{\text{o}}} \right)}} \left( \omega \right),$$

where ${\eta }^{(\text{w}/\text{o})}\left(\omega \right)$ is also an input supplied by FDTD simulations with semi-infinite quartz substrate. We evaluated the above equation (Eq. 10) for a range of frequencies and substrate thicknesses using the same PMR parameters as before and the resulting intensity map is plotted in Fig. 3a. Clearly, we were able to reproduce the dispersion branches including their shape and the abrupt gap that disects them at a specific frequency, corresponding to the substrate thickness equal to half-multiples of wavelength in quartz (indicated by white dotted lines).

The origin of the gap is revealed by inspection of Eq. (9): the enhancement of the excitation amplitude $j\left(\omega \right)$, and therefore also of the THz magnetic field, brought by the quartz/gold interface can be broken down into two distinct contributions: (i) the incident field factor (${E}_{\text{in}}^{(\text{w})}\left(\omega \right)$), corresponding to a standing wave formed in the substrate by the illuminating beam and (ii) the array feedback factor (${E}_{\text{re}}^{(\text{w})}\left(\omega \right)$), representing the interaction of the antenna array with itself (see also Fig. 2b). To analyze the effects of these two factors on the magnetic field intensity enhancement ${\eta }^{(\text{w})}\left(\omega \right)$ we recalculated the intensity map from Fig. 3a for the two factors separately, i.e. we kept one, omitted the other one and vice versa. The intensity map based on just the incident field factor (Fig. 3b) shows broad maxima alternating with broad minima, depending on whether the forward and the back-reflected waves interfere constructively or destructively. The array feedback factor (Fig. 3c), on the other hand, yields a strikingly sharp response, indicating a strong positive feedback between the antenna array and its own radiation. Most importantly, maxima of the array feedback factor fall into minima of the incident field factor and vice versa. The reason behind this mutual misalignment can be traced to the phase shift between the radiation emitted by the antenna (${E}_{\downarrow }\left(\omega \right)$) and the incoming field driving it (${E}_{\text{dr}}\left(\omega \right)$). Frequency dependence of this phase shift (blue line) is plotted in Fig. 3d. When the array is in resonance—which occurs when the amplitude of the emitted wave (red line) is at its maximum—this phase shift is exactly $\pi$ and the optimum condition for constructive interference, which translates into a maximum array feedback factor, coincides with a zero in the incident field factor (${E}_{\text{in}}^{(\text{w})}\left(\omega \right)/{E}_{\text{in}}^{(\text{w}/\text{o})}\left(\omega \right)$), which manifests itself as a sudden gap in the dispersion branch. By deviating slightly from the resonance, the incident field factor is no longer zero and its product with the dominant array feedback factor yields a considerable response at both sides of the gap. As we move away from the resonance, the magnetic field intensity enhancement gradually fades. This is partly due to the inability of the incident field factor to compensate for the decline in the array feedback factor, but also due to the vanishing of the magnetic field generated by the currents flowing within the antenna array.

The model presented here not only perfectly reproduces the PMR response obtained by numerical simulations (Fig S1), but also provides a clear explanation for the sharpness of this resonance: the antenna array and the back-reflector form a Fabry-Pérot resonator that allows a build-up of energy within the device. In contrast to standard cavity resonators encountered in EPR experiments, the active volume is not in the center of the resonator, but at its boundary. In addition to participating in the Fabry-Pérot oscillations that enhance the strong positive feedback mentioned above, the antenna array also enhances the magnetic field by shaping and funneling the currents at the level of the individual antennas. The synergy between these two effects makes our PMR device a promising platform for ultrasensitive THz EPR spectroscopy of thin films. Our model is not limited to THz magnetic field enhancement but can also be used to improve the response of cavity-enhanced metasurfaces proposed for a wide range of applications^30–33^.

### Polarization dependence

**Figure 4:**
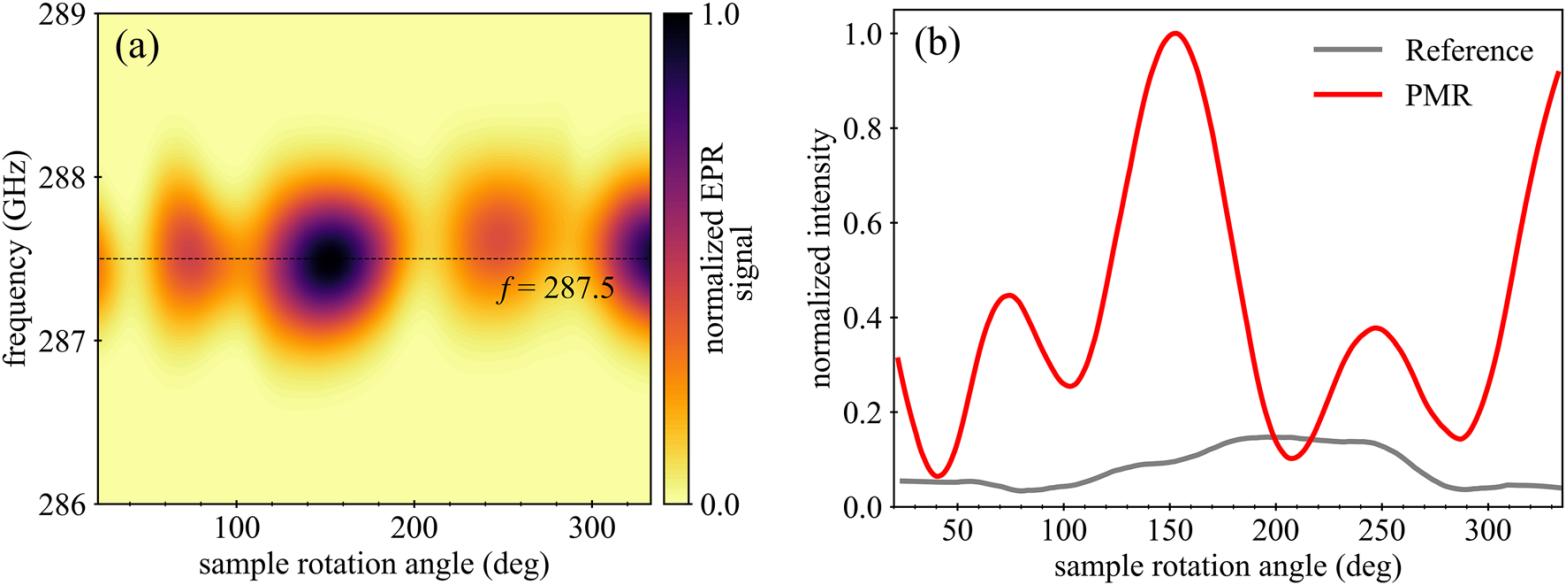
(**a**) Experimental map of the magnetic resonance of TEMPOL radical, centred at the resonant frequency of the PMR (287.5 GHz, see text), as a function of the frequency and of the angle between the PMR and the incident electric field vector, measured in a constant magnetic field of 10.22 T and a temperature of 10 K. (**b**) Angular profile taken at the frequency of 287.5 GHz for the sample deposited on the PMR (red) and on a bare quartz substrate (grey, reference).

In THz EPR, the quasi-optic setup is designed to limit the radiation power at the detector in order to measure on a “zero” background, making the relative signal intensity change bigger. Specifically, the THz radiation that is generated by the source is linearly polarized and after interacting with the magnetic sample, which absorbs preferentially either the left or right circular polarization, this radiation presents an elliptical shape. Through a series of polarizers and Faraday rotator elements, it is possible to separate the radiation with polarization parallel to the incoming radiation and perpendicular to that. The latter is the only component registered by the detector, as it contains the information of the sample investigated. Such a scheme is known as induction mode detection^22,34,35^. In our experiment, the diabolo antennas that make up the PMR are only activated when the polarization of the incident THz electric field is parallel to the long axis of the antenna (see Fig. 1)^36^. The scattered electric field is also parallel to the long axis of the antenna, which makes it difficult to detect using the induction mode detection scheme. Therefore, the optimal orientation of the incident polarization with respect to the diabolo antenna is not obvious a priori. This issue motivated us to investigate the influence of the THz polarization with respect to the orientation of the diabolo antennas on the EPR signal intensity. The magnetic sample was deposited on the PMR and its THz EPR spectrum was recorded by rotating the sample in situ with a piezoelectric rotator. For the experiment, TEMPOL radical (4-hydroxy-2,2,6,6-tetramethylpiperidine-1-oxyl) was diluted 5% w/w in a polymer matrix of PMMA (poly(methyl methacrylate)) and the mixture spin-coated on top of the PMR. PMMA is known to form smooth films and the concentration of TEMPOL was kept rather low for two reasons: (i) too high concentrations can have a detrimental effect on the quality of the spin-coated films and (ii) to limit the dipolar interactions between the radicals. The film produced covered the entire substrate area homogeneously with a resulting thickness of 330 ± 10 nm. The resonance frequency of the PMR was experimentally determined in our previous work to be 287.5 GHz^23^. By setting an external static magnetic field of 10.22 T, due to the Zeeman splitting, the magnetic resonance of the TEMPOL radical also occurs at 287.5 GHz. In this condition we can observe the enhancement of the EPR signal driven by the PMR. The THz EPR intensity maps were recorded as a function of angle (25–335°) and frequency (286–289 GHz), at a constant temperature of 10 K. The resulting map is shown in Fig. 4a and a horizontal 1D profile of the map at 287.5 GHz is shown in Fig. 4b. The angular dependence of the signal intensity exhibits two local maxima at 75 and 245° and two absolute maxima at 153° and around 335°. The last peak falls in the blind spot of the piezoelectric rotator (335°–25°). A 180° periodicity is found between each pair of maxima, while a 90° periodicity is found between the relative and absolute maxima. The experiment was repeated on an analogue sample prepared on a bare quartz substrate, without diabolo antennas. In this case, the overall signal is weaker and there is no clear angular dependence (Fig. 4b).

**Figure 5:**
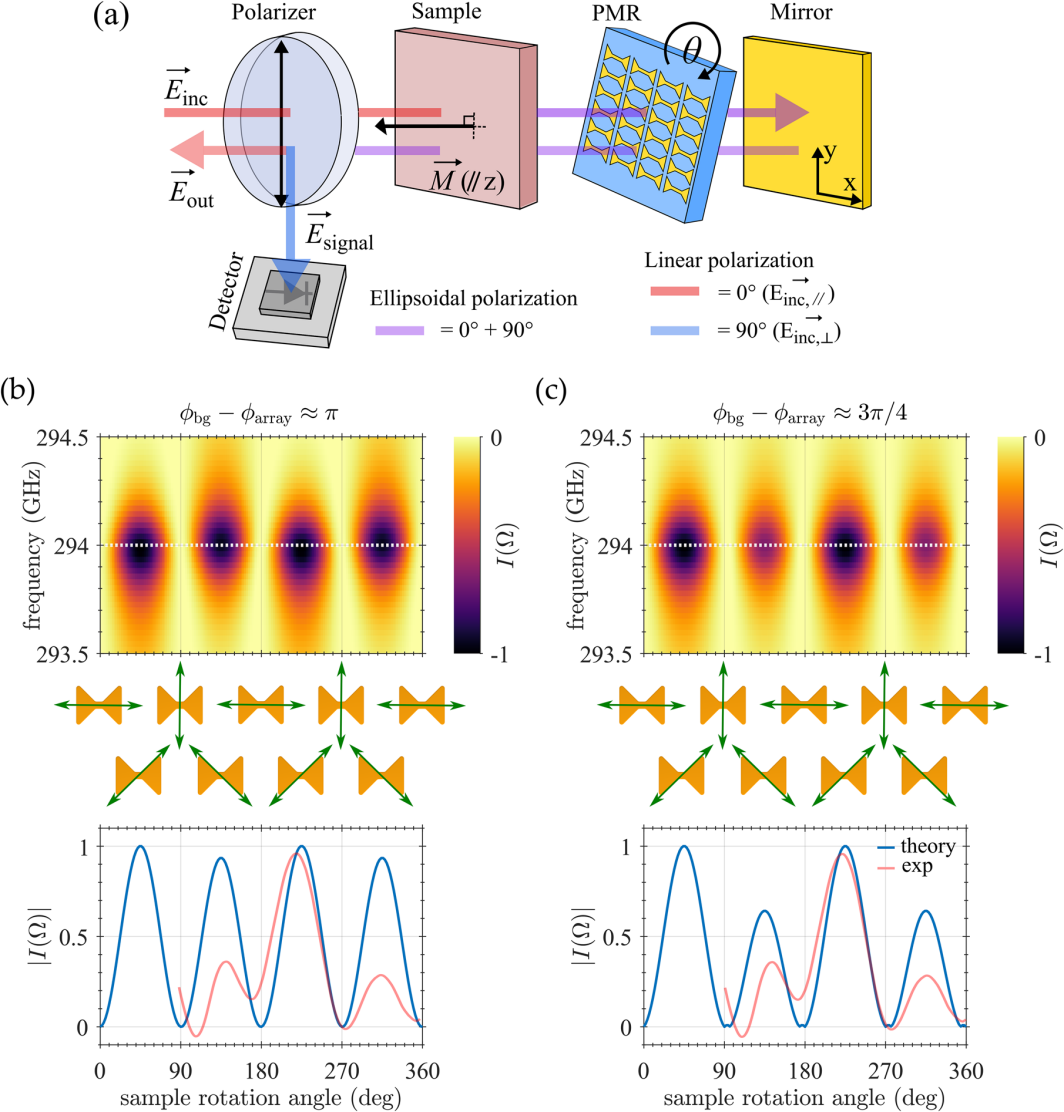
(**a**) Schematics of the simplified optical path considered in our theoretical model. After passing the first polarizer, radiation interacts with the sample and is reflected from the PMR. Before it reaches the detector, it once again passes through the magnetic layer and is filtered by the second polarizer. Sample, PMR and mirror are separated for clarity. (**b**) Intensity map of the EPR signal as a function of the frequency and the angle formed by the long axis of the diabolo antennas and the plane of polarization of the illuminating wave. The map was calculated using our theoretical model, with input parameters based on simulations of a PMR exhibiting optimal performance. The angular profile at the bottom corresponds to a cut along the white dotted line in the intensity map and is shown together with the experiment for comparison. The pictograms show the mutual orientation of the diabolo antenna and the incident electric field vector for several significant values of the angle $\theta$. (**c**) Same as in (**b**), except the phase shift ${\phi }_{\text{bg}}-{\phi }_{\text{array}}$ between the field amplitudes ${r}_{\text{bg}}$ and ${r}_{\text{array}}$ was artificially set to 3$\pi /4$. Apparently, the departure from the ideal resonance of the PMR can lift the conditions that prevented Faraday effect from manifesting and break the original four-fold symmetry of the angular profile.

In order to explain the experimental results, we developed an approximate semi-analytical model that incorporates both the Faraday effect from the magnetic sample and the anisotropic response of the antenna array, taking into account the induction mode detection scheme used in the experiment. To keep our model intelligible and tractable, we adopted a simplified version of the quasi-optical path encountered in our experimental setup and shown in Fig. 5a: after passing the polarizer, the incident beam interacts with the sample and, as a result of the Faraday effect, its plane of polarization is rotated. The radiation then passes through the PMR and is reflected by the mirror (here separated from the PMR for clarity). On the way back, the radiation interacts again with the PMR and the sample, and is then filtered by the polarizer, so that only the perpendicular component of the polarization reaches the detector. Each object along this path is represented by a Jones matrix, which fully captures the individual effects on the intensity and polarization state of the passing THz radiation. Denoting $\theta$ the angle between the long axis of the diabolo antennas and the plane of polarization of the THz radiation enforced by the first polarizer, the electric field vector of the wave incident on the sample reads

11 $$\vec{E}_{{{\text{inc}}}} = \left[ {\begin{array}{*{20}c} {\cos \theta } \\ {\sin \theta } \\ \end{array} } \right].$$

The Faraday effect can be linked to the appearance of non-zero off-diagonal elements in the dynamic magnetic permeability, leading to a rotation of the plane of polarization due to a difference in the refractive index perceived by waves of opposite handedness. We adopt the following form of the magnetic permeability tensor

12 $$\mathop{\mu }\limits^{\leftrightarrow} = \left[ {\begin{array}{*{20}c} {1 + \chi \left( \omega \right)} & {\quad \mp \,i\chi \left( \omega \right)} & {\quad 0} \\ { \pm \,i\chi \left( \omega \right)} & {\quad 1 + \chi \left( \omega \right)} & {\quad 0} \\ 0 & {\quad 0} & {\quad 1} \\ \end{array} } \right],$$

where the dynamic magnetic susceptibility $\chi \left(\omega \right)$ describes the material properties of the paramagnetic sample. For materials with a single magnetic dipole transition, such as the TEMPOL radical, $\chi \left(\omega \right)$ can be approximated by a simple Lorentzian function with a matching relaxation time $\tau$ and a resonance frequency ${\omega }_{\text{m}}$ dependent on the static magnetic field

13 $$\chi \left( \omega \right) = \frac{{\kappa \omega_{{\text{m}}} }}{{\omega_{{\text{m}}}^{2} - \omega^{2} - 2i\omega /\tau }},$$

where the amplitude $\kappa$ scales with the density of spin centers within the material that can be excited. Depending on their handedness, electromagnetic waves propagating in such a medium along the $z$-axis (i.e. in the direction of the static magnetic field) experience a different refractive index.^37^ In the Jones matrix formalism, this translates to

14 $$\mathop{M}\limits^{\leftrightarrow} = \frac{1}{2}\left[ {\begin{array}{*{20}c} 1 & {\quad 1} \\ i & {\quad - i} \\ \end{array} } \right]\left[ {\begin{array}{*{20}c} {e^{i\alpha } } & {\quad 0} \\ 0 & {\quad 1} \\ \end{array} } \right]\left[ {\begin{array}{*{20}c} 1 & {\quad - i} \\ 1 & {\quad i} \\ \end{array} } \right] = e^{{\frac{i\alpha }{2}}} \left[ {\begin{array}{*{20}c} {\cos \frac{\alpha }{2}} & {\quad \sin \frac{\alpha }{2}} \\ { - \sin \frac{\alpha }{2}} & {\quad \cos \frac{\alpha }{2}} \\ \end{array} } \right],$$

where the exponential factor

15 $$e^{i\alpha } \approx e^{{i\frac{\omega }{c} \chi \left( \omega \right) d}}$$

captures both the difference in the accumulated phase and the decrease in amplitude experienced by those two types of waves in a thin magnetic sample of thickness $d$. Similarly, the PMR can be effectively modelled as an anisotropic reflective layer,

16 $$\mathop{R}\limits^{\leftrightarrow} _{{{\text{PMR}}}} = \left[ {\begin{array}{*{20}c} {r_{x} } & {\quad 0} \\ 0 & {\quad r_{y} } \\ \end{array} } \right] = \left[ {\begin{array}{*{20}c} {r_{{{\text{bg}}}} + r_{{{\text{array}}}} + \xi } & {\quad 0} \\ 0 & {\quad r_{{{\text{bg}}}} } \\ \end{array} } \right],$$

where the *x*-direction is defined to lie along the long diabolo axis (see Fig. 1), ${r}_{\text{array}}$ stands for the amplitude of the wave emitted by the antenna array, ${r}_{\text{bg}}$ represents the Fresnel coefficient of the background (i.e. the quartz substrate with the metallic back-reflector), and $\xi$ acts as a perturbation to ${r}_{\text{array}}$ due to the interaction of the antenna array with the magnetic material. The last missing ingredient is the Jones matrix representation of the second polarizer, $\mathop{P}\limits^{\leftrightarrow} \left( \theta \right)$. The requirement for its orthogonality with respect to the incident electric field yields

17 $$\mathop{P}\limits^{\leftrightarrow} \left( \theta \right) = \left[ {\begin{array}{*{20}c} {\sin^{2} \theta } & {\quad - \sin \theta \cos \theta } \\ { - \sin \theta \cos \theta } & {\quad \cos^{2} \theta } \\ \end{array} } \right].$$

Finally, the electric field vector of the wave reaching the detector, ${\vec{E}}_{\text{signal}}$, is obtained by stacking all the above Jones matrices in the proper order

18 $$\vec{E}_{{{\text{signal}}}} = \mathop{P}\limits^{\leftrightarrow} \left( \theta \right) \mathop{M}\limits^{\leftrightarrow} \mathop{R}\limits^{\leftrightarrow} _{{{\text{PMR}}}} \mathop{M}\limits^{\leftrightarrow} \vec{E}_{{{\text{inc}}}} = \frac{1}{2}e^{i\alpha } \left[ {\sin 2\theta \left( {r_{x} - r_{y} } \right) + \sin \alpha \left( {r_{x} + r_{y} } \right)} \right]\left[ {\begin{array}{*{20}c} {\sin \theta } \\ { - \cos \theta } \\ \end{array} } \right] .$$

The measured EPR signal is then simply proportional to the square modulus of the electric field, namely

19 $$I_{{{\text{signal}}}} \left( \theta \right)\sim \left| {\vec{E}_{{{\text{signal}}}} } \right|^{2} = \frac{1}{4}e^{{ - 2 {\text{Im}}\left\{ \alpha \right\}}} \left[ {\sin^{2} 2\theta \left| {r_{x} - r_{y} } \right|^{2} + \left| {\sin \alpha } \right|^{2} \left| {r_{x} - r_{y} } \right|^{2} + 2\sin 2\theta {\text{Re}}\left\{ {\sin \alpha \left( {r_{x} + r_{y} } \right)\left( {r_{x}^{*} - r_{y}^{*} } \right)} \right\}} \right] .$$

To fully reproduce the experimental conditions, it is important to consider the lock-in detection, which is done by modulating the magnetic field at the frequency $\Omega$. To reflect this in our model, we should keep only those terms that directly depend on the modulation frequency $\Omega$. The static magnetic field has an impact only on the parameters $\xi$ and $\alpha$, and, because the THz magnetic radiation is weakly interacting with the magnetic material, we can neglect any terms other than those linear in $\xi$ or $\alpha$. Under these conditions, Eq. (19) reduces to

20 $$I_{{{\text{signal}}}} \left( {\theta ,\Omega } \right) \approx - \frac{1}{2}{\text{Im}}\,\left\{ {\alpha \left( \Omega \right)} \right\} \left| {r_{{{\text{array}}}} } \right|^{2} + \frac{1}{2}\sin^{2} 2\theta \,{\text{Re}}\left\{ {\xi \left( \Omega \right) r_{{{\text{array}}}}^{*} } \right\} + \frac{1}{2}\sin 2\theta \,{\text{Re}}\left\{ {\alpha \left( \Omega \right) r_{{{\text{array}}}}^{*} \left( {2r_{{{\text{bg}}}} + r_{{{\text{array}}}} } \right)} \right\} + \cdots .$$

Looking at the above expression, one immediately notices the differences in angular dependences of the three leading terms that might, in principle, produce the experimentally measured angular profile plotted in Fig. 4b. To assess this hypothesis, we need to evaluate Eq. (18) using plausible values for the parameters $\xi$ or $\alpha$ (the rest is supplied by numerical simulations). While $\alpha$ was already defined in terms of the various parameters characterizing the magnetic layer (see Eq. 15), $\xi$ remains to be specified. Invoking the theorem describing energy conservation in lossy media^38^, we can link the change in the absorbance, $\Delta A$, due to the interaction between the antenna array and the magnetic material, to the average magnetic field intensity enhancement $\eta \left(\omega \right)$ provided by the antenna array

21 $$\Delta A = \frac{{\frac{1}{2}\omega \mu_{0} {\text{ Im}}\left\{ {\chi \left( \omega \right)} \right\}\int_{V} {dV \left| {\vec{H}_{{ \circlearrowleft }} } \right|^{2} } }}{{\frac{1}{2}S\mu_{0} c\left| {\vec{H}_{0} } \right|^{2} }} = \frac{\omega }{c}d {\text{Im}}\left\{ {\chi \left( \omega \right)} \right\} \eta .$$

Here the denominator represents the radiant flux illuminating the surface area $S$ of the sample and the subscript $\circlearrowleft$ indicates that only a portion of the magnetic field with the proper handedness can induce the magnetic resonance effect. Realizing that an increase in the absorbance should be accompanied by a decrease in the amplitude of the wave emitted by the antenna array, we also find that

22 $$\Delta A = \left| {r_{{{\text{array}}}} } \right|^{2} - \left| {r_{{{\text{array}}}} + \xi } \right|^{2} \approx - 2{\text{Re}}\left\{ {\xi r_{{{\text{array}}}}^{*} } \right\}.$$

At resonance, the complex amplitude $\xi$ is expected to be opposite in phase to ${r}_{\text{array}}$ (to maximize the rate at which energy is drained from the antenna array), and the above equation becomes

23 $$\Delta A = - 2\left| \xi \right|\left| {r_{{{\text{array}}}} } \right|{\text{ Re}}\left\{ {e^{i\pi } } \right\} = 2\left| \xi \right|\left| {r_{{{\text{array}}}} } \right|.$$

Finally, by combining Eqs. (21) and (23), we obtain an estimate for $\xi$ in terms of the average magnetic field intensity enhancement $\eta$ and other various parameters

24 $$\left| \xi \right| = \frac{\omega }{{2c\left| {r_{{{\text{array}}}} } \right|}}d {\text{Im}}\left\{ {\chi \left( \omega \right)} \right\} \eta .$$

Inspection of the above expression reveals that the antenna array should boost the EPR signal roughly by a factor of $\eta$ compared to a situation when only the Faraday effect takes place.

At this point we have all the ingredients to simulate the angular dependence of the EPR signal, ${I}_{\text{signal}}\left(\theta ,\Omega \right)$. We set the value of $\eta$ to 10, which corresponds to a ratio between $\xi$ and $\alpha$ close to 10. This value is consistent with our previous findings^23^. The resulting intensity map is shown in Fig. 5b, together with a 1D section through $\theta$ at the PMR resonant frequency. The calculated profile shows no variation in the height of the four peaks, which correspond to the four optimal orientations of the antenna array with respect to the polarizers. It should be noted that the resonant frequency from the simulations is slightly higher than the experimental values by a few GHz, which is a marginal difference and can be attributed to uncertainty in the refractive index of the materials or substrate thickness. The symmetry-breaking term associated with the Faraday effect seems to have almost no impact on the angular profile, despite the rather conservative choice of $\eta$. The reason behind this unexpected outcome is revealed by a detailed analysis of the relevant quantities: Denoting ${\phi }_{\text{bg}}$ and ${\phi }_{\text{array}}$ as the phases of the respective field amplitudes ${r}_{\text{bg}}$ and ${r}_{\text{array}}$, the third term in Eq. (20) can be rewritten as

25 $$\frac{1}{2}\sin 2\theta \,{\text{Re}}\left\{ {\alpha \left( \Omega \right) r_{{{\text{array}}}}^{*} \left( {2r_{{{\text{bg}}}} + r_{{{\text{array}}}} } \right)} \right\} = \frac{1}{2}\sin 2\theta \,{\text{Re}}\left\{ {\alpha \left( \Omega \right) \left[ {\left| {r_{{{\text{array}}}} } \right|^{2} + 2\left| {r_{{{\text{bg}}}} } \right|\left| {r_{{{\text{array}}}} } \right|e^{{i\left( {\phi_{{{\text{bg}}}} - \phi_{{{\text{array}}}} } \right)}} } \right]} \right\} .$$

Our simulations show that the field amplitudes ${r}_{\text{bg}}$ and ${r}_{\text{array}}$ are more or less opposite in phase, i.e. ${\phi }_{\text{bg}}-{\phi }_{\text{array}}\approx \pi$. From the energy dissipation viewpoint, this means that when in resonance, the PMR draws power from the source at the highest rate and, for that to happen, the phase of the driving and the scattered fields must be opposite. Meanwhile, $\alpha$ is purely imaginary at the frequency of the magnetic dipole transition (cf. the expression for $\chi \left(\omega \right)$ given by Eq. (13) when $\omega ={\omega }_{\text{m}}$). Therefore, the whole expression in the braces is imaginary as well and its role in the angular dependence of the EPR signal turns out to be marginal.

It should be highlighted that such a model is idealized, in the sense that the values of the field amplitudes ${r}_{\text{bg}}$ and ${r}_{\text{array}}$ are based on perfect simulations with optimized geometry, therefore neglecting minor defects in the fabrication of the PMR or in the experimental setup. This is evidenced by the relatively modest plasmon-assisted enhancement of the EPR signal observed in our previous experimental work^23^, which resulted in a factor 30 instead of the theoretically predicted value 160. It is therefore possible that the phase shift between ${r}_{\text{bg}}$ and ${r}_{\text{array}}$ is not exactly $\pi$. To explore this scenario, we recalculated the intensity map using a different value for ${\phi }_{\text{bg}}-{\phi }_{\text{array}}$, namely $3\pi /4$. The resulting angular profile is plotted in Fig. 5c and breaking of its original four-fold symmetry is now clearly visible. This description is not an irrefutable proof of our hypothesis designating the Faraday effect as the party responsible for the peculiar angular profile of the measured EPR signal: there are other potential candidates that are not included in our model but could act as sources of anisotropy (e.g. sample tilt, standing waves in the sample holder, grease securing the sample to the holder, manufacturing defects). At the same time, we cannot exclude the Faraday effect as the main culprit, since our model is based on several assumptions, such as the optimal functioning of the PMR, which are not necessarily fulfilled in reality. A more complex model and additional experimental data are required to make a final judgement on this matter.

To sum up this part, we have carried out THz EPR experiments to investigate the response of the PMR as a function of the orientation. To disentangle the contribution of the antennas anisotropy from the detection scheme used for the THz EPR experiments, we have developed a semi-analytical model. This model enables us to analyse in detail the polarization modifications induced by the PMR as well as the effect of the individual experimental setup components: polarizer, magnetic sample, and back-reflector. Indeed, although the efficiency of the PMR is maximum when the linearly polarized radiation is parallel to the antenna’s long axis (0°), the greatest EPR signal enhancement occurs when the polarization is at 45° due to the detection scheme used in THz EPR spectroscopy. The asymmetry of the experimentally observed peaks instead remains an open point, which requires more experiments.

## Conclusions

In this study, we advanced the understanding of the THz plasmonic metasurface resonator previously used to enhance the magnetic signal in THz EPR^23^, by developing two comprehensive semi-analytical models to explain: (i) the origin of its strong magnetic resonance and (ii) its anisotropic behaviour. The first model successfully reproduced the magnetic resonance response, revealing the synergistic interplay between the metasurface resonance and Fabry-Pérot standing waves in the substrate formed by a back-reflector. The metasurfaces are activated by both the incident radiation and the radiation emitted from the metasurfaces, forming standing waves with a very high Q-factor. Therefore, the metasurface feeds itself, entering into a sort of self-driving mode that increases its overall response. This model also explains the broken dispersion branch, which is due to constructive interference between the driving electric field and the emitted field of the metasurface.

The second model elucidated the influence of the anisotropy and polarization of the antennas constituting the metasurface on the overall response of the plasmonic metasurface resonator. This model, which takes into account the commonly used detection scheme of THz EPR, showed that the optimal EPR signal enhancement occurs at a 45° polarization angle, although the individual metasurface efficiency is maximal for linearly polarized radiation aligned with the long side of the antennas. The model also suggests that the asymmetry observed experimentally is most likely due to imperfections of the real experimental setup, rather than to some degree of rotation of the polarization induced by the magnetic sample through Faraday rotation. This model clearly indicates the need to use a different experimental scheme that allows the detection of radiation that is linearly polarized parallel to the incident radiation.

Overall, these two models provide a complete playground to study the properties of THz plasmonic magnetic metasurface resonators. Thus, this study allows the design of new metasurfaces with even stronger magnetic field enhancement for sensing applications, as well as the control of their polarization to optimize the experimental conditions.

## Experimental

### CST Studio simulations

The software CST Microwave Studio (Dassault Systèmes) was used. The simulations were performed with the Time Domain Solver, using a linearly polarized Gaussian beam source with a radius of 1.5 mm and normal incidence with respect to the PMR surface. A 7 × 7 gold antennas array was considered, together with a quartz substrate and a back-reflector (the parameters are reported in Fig. 1a). The frequency stepped from 200 to 400 GHz with steps of 2 GHz, while the thickness of the substrate was swept between 150 and 1200 µm with steps of 15 µm. The square of the maximum of the in-plane magnetic near field was detected at a distance of 10 nm above the antennas’ plane.

### Lumerical simulations

Optical response of diabolo antennas that served as an input to our semi-analytical models was obtained using Ansys Lumerical Finite-Difference Time-Domain (FDTD) software package. Adopting the Total-Field Scattered-Field (TFSF) illumination configuration, we monitored the polarization current induced within a single diabolo antenna and exported it into Matlab for further postprocessing. Magnetic field enhancement and far field radiation amplitude were calculated from the current distribution using Green’s function formalism. The effects of mutual interaction between antennas and reflections from interfaces between media not present within the single antenna simulation (e.g. the gold back-reflector) were introduced by appropriately scaling the amplitude of the current distribution at each antenna site. This scaling factor was determined from our in-house developed approximate semi-analytical model enabling accurate evaluation of inter-antenna coupling in large scale arrays.

### Sample preparation

The chemicals 4-hydroxy-2,2,6,6-tetramethylpiperidine-1-oxyl (TEMPOL, assay ≥ 98%, purchased from Fluka Analytical), and poly(methyl methacrylate), PMMA, (MW ≈ 350 000 by GPC, density: 1.17 g mL^−1^, purchased from Sigma Aldrich) were used without further treatments. The sample preparation follows exactly what reported in our previous article^23^ and is hereafter summarised. A solution of 5% (w/w) TEMPOL in 50 gL^-1^ of PMMA in chlorobenzene was prepared and used for spin-coating the PMR. The PMR was previously cleaned in subsequent ultrasonic baths of isopropanol and acetone, followed by a gentle CO_2_ stream (Applied Surface Technology Snowjet) while heating it at 100 °C. The rotation rate for the spin-coating was 2000 rpm for 1 min. The thickness was obtained by profilometry (Dektak Stylus Profiler, Bruker).

### THz EPR experiments

A home-built spectrometer was used and its complete description can be found in reference^34^. The measurements were done in induction mode (as explained in section “Polarization Dependence”). The sample holder was equipped with a piezoelectric rotator that allows rotating the sample in situ. The experimental frequency vs angle map was recorded using a field modulation of 3 mT of amplitude and 30 kHz of frequency. The angle was measured every 2 degrees, with the exclusion of the blind spot of the piezoelectric rotator (ca. 50 degrees, from 335 to 25 deg). The THz radiation frequency was swept with 5 s per scan and averaging 3 spectra each time. The signal was processed by fitting the individual curves with a Gaussian derivative model and integrating the fitted curves. The plot of the integrated signal instead of the derivative shape provides the easiest visualization of the signal enhancement and angular dependence. Data visualization and processing was done using the NumPy, SciPy and Matplotlib libraries in Python^39–41^.

### Supplementary Information

Supplementary Information.

## Data Availability

The authors declare that all data supporting the findings of this study are available within the paper and its Supplementary Information files. Additional data related to this paper are available from the corresponding authors upon reasonable request.
