# Supplementary material for: Terahertz magnetic response of plasmonic metasurface resonators: origin and orientation dependence
Source: Sci Rep. 2024 Jul 3;14:15305. doi: 10.1038/s41598-024-65804-9 (PMC11222506; doi:10.1038/s41598-024-65804-9)
Supplement: Supplementary file 1 — Supplementary Information. [file 41598_2024_65804_MOESM1_ESM.docx]

**SUPPORTING INFORMATION**

**Terahertz Magnetic Response of Plasmonic Metasurface Resonators: Origin and Orientation Dependence**

Lorenzo Tesi,^*,+,a^ Martin Hrtoň,^+,b^ Dominik Bloos,^a^ Mario Hentschel,^c^ Tomáš Šikola,^b^ Joris van Slageren^*,a,d^

**Keywords**: magnetic metasurface; terahertz; cavity-enhanced; Fabry-Pérot; electron paramagnetic resonance.

^a^ Institute of Physical Chemistry, University of Stuttgart, Pfaffenwaldring 55, 70569 Stuttgart, Germany. E-mail: [lorenzo.tesi@ipc.uni-stuttgart.de](mailto:lorenzo.tesi@ipc.uni-stuttgart.de)

^b^ Institute of Physical Engineering and Central European Institute of Technology, Brno University of Technology, Technická 2, 61669 Brno, Czech Republic.

^c^ 4th Physics Institute and Research Center SCoPE, University of Stuttgart, Pfaffenwaldring 57, 70569 Stuttgart, Germany.

^d^ Center for Integrated Quantum Science and Technology, University of Stuttgart, Germany. E-mail: [slageren@ipc.uni-stuttgart.de](mailto:slageren@ipc.uni-stuttgart.de)

^+^These authors contributed equally.


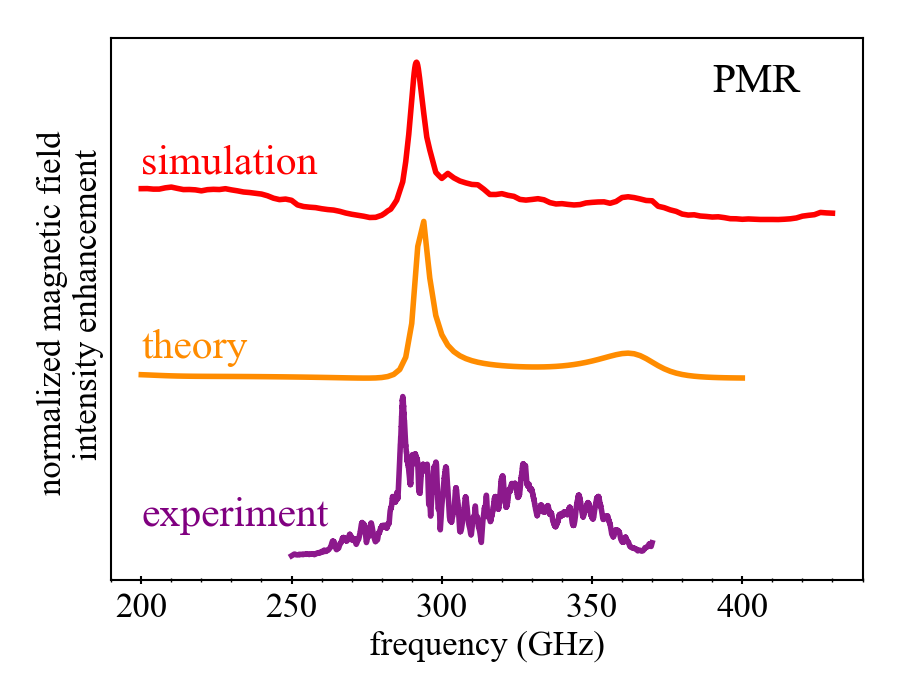


**Figure S1.** Frequency dependence of the magnetic field intensity enhancement obtained by CST Studio numerical simulations (top), by applying the semi-analytical model that is described in the main article (middle), and by high frequency electron paramagnetic resonance spectroscopy experiments (bottom) obtained by probing the magnetic resonance of a thin film of a radical sample dispersed in a polymeric matrix (more details in Tesi *et al.* 10.1002/smtd.202100376). The experimental response includes the profile of the terahertz source and the standing waves from the optical path and sample-holder cavity. Also, the resonant peak is slightly shifted to lower frequency because of the thin film onto the PMR.


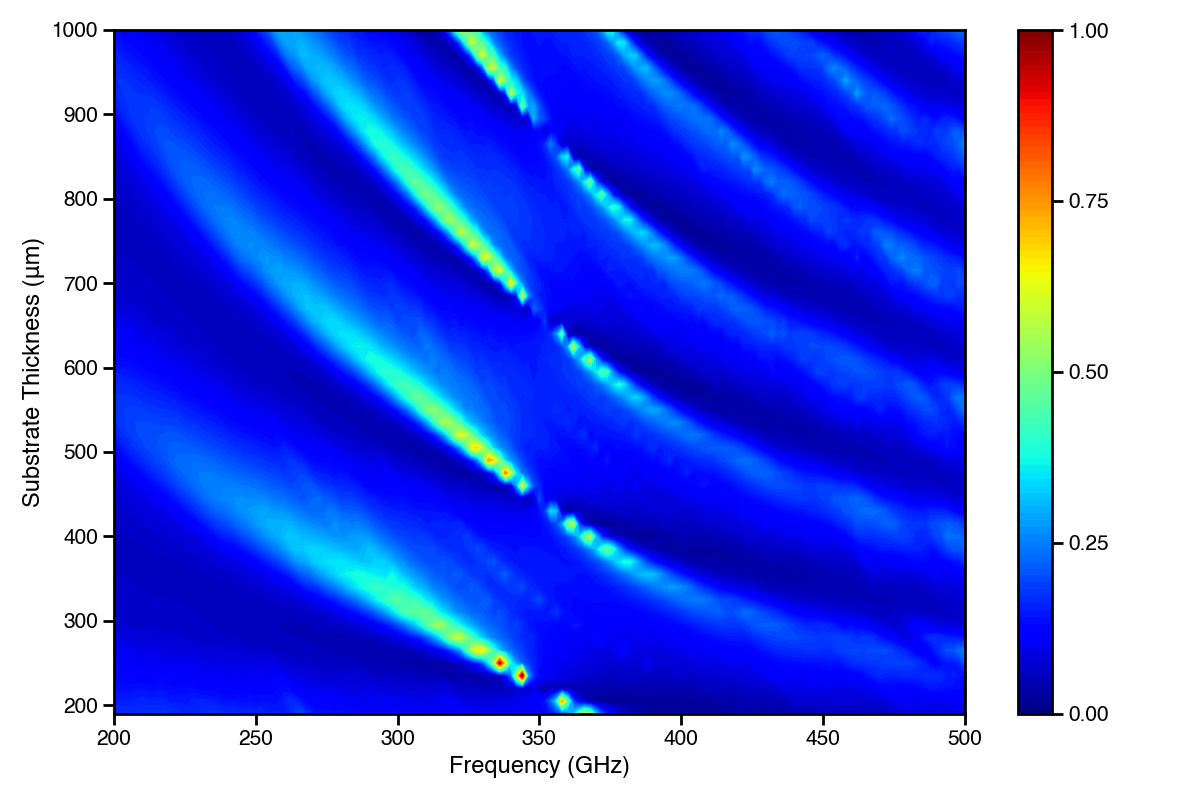


**Figure S2.** Map of the magnetic field intensity enhancement of the PMR as a function of the frequency and the substrate thickness, performed using CST Microwave Studio numerical simulations. The following parameters were used: antenna length 168 μm, antenna height 100 μm, bridge length 29 μm, bridge width 17 μm, antenna thickness 140 nm, antennas gap 330 μm.


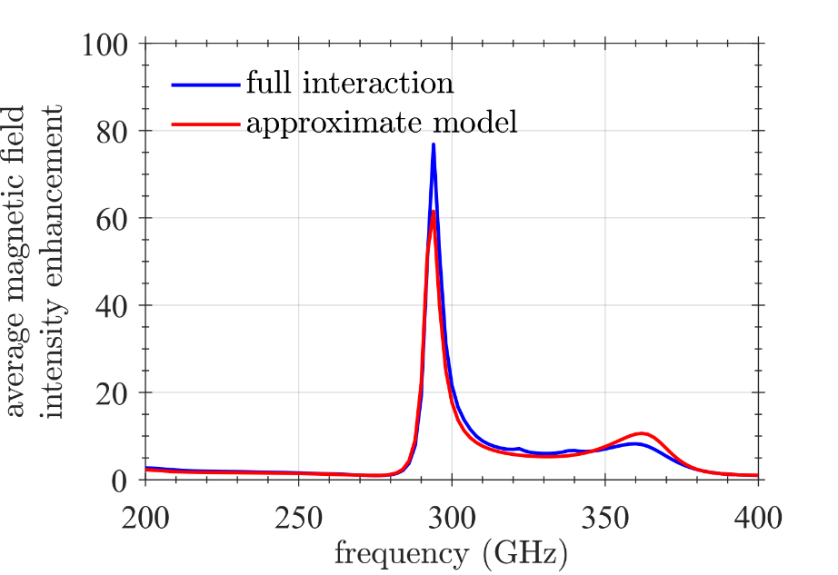


**Figure S3.** Blue line represents the magnetic field intensity enhancement calculated by taking fully into account the interaction between each diabolo antenna and the gold back-reflector (employing Green’s function formalism). The red line corresponds to the magnetic field intensity enhancement obtained from our approximate model, in which the back-action experienced by an antenna array when it interacts with its own radiation is modelled using a Fabry-Pérot resonator. The latter approach correctly predicts both the overall shape and the spectral position of the sharp resonance, demonstrating the validity of our approximate model treating the whole antenna array as a single cohesive entity.
